# Effect of fumaric acid in combination with *Asparagopsis taxiformis* or nitrate on in vitro gas production, pH, and redox potential

**DOI:** 10.3168/jdsc.2022-0259

**Published:** 2023-07-13

**Authors:** M. Thorsteinsson, M. Maigaard, P. Lund, M.R. Weisbjerg, M.O. Nielsen

**Affiliations:** Department of Animal and Veterinary Sciences, Aarhus University, AU Viborg–Research Centre Foulum, DK-8830 Tjele, Denmark

## Abstract

Reduction in enteric methane (CH_4_) emissions from cattle can be achieved through use of feed additives, which often results in increased emission of hydrogen (H_2_). The objective of this study was to investigate in vitro effects of a known hydrogen sink, fumaric acid, in combination with either of 2 methane inhibitors, the macroalga *Asparagopsis taxiformis* or nitrate, on CH_4_ and H_2_ production, feed degradability, pH, and redox potential. A corn silage (0.5 g; control) was incubated in buffered rumen fluid with the addition of 0.025 g of nitrate (Nit), 0.025 g of dried *A. taxiformis* (Asp), 0.025 g of nitrate + 0.025 g of fumaric acid (Nit+Fum), or 0.025 g of dried *A. taxiformis* + 0.025 g of fumaric acid (Asp+Fum). Accumulated gas production was determined using the Ankom^RF^ system equipped with airtight gasbags. There were 9 replicates per treatment with 3 replicates per treatment stopped after 24, 36, and 48 h of incubation. The amount of undegraded feed was determined by filtration. Gas composition was determined by gas chromatography. Degradable dry matter, degradable organic matter, pH, redox potential, and gas production data were analyzed using a mixed model. Asp and Asp+Fum reduced CH_4_ production by 98% or greater at all incubation times, whereas Nit and Nit+Fum reduced CH_4_ production (mL of CH_4_/g of dry matter) by 52% to 63% compared with the control. Hydrogen was only detectable in gas from Asp and Asp+Fum treatments, with no difference in H_2_ production between the 2 treatments. The treatments had only minor effects on redox potential in the fermented rumen fluid, and pH was lowest for treatments including *A. taxiformis.* In conclusion, both *A. taxiformis* and nitrate reduced CH_4_ production. Fumaric acid in combination with *A. taxiformis* did not reduce H_2_ production, and treatments including nitrate did not result in any detectable levels of H_2_. Future dose-response in vitro studies will contribute to investigating the potential of fumaric acid as a hydrogen sink during CH_4_ mitigation.

## Highlights

• Fumaric acid had no effect on hydrogen production when methane was inhibited with *A. taxiformis*.
• Hydrogen was not detectable when methane was inhibited with nitrate.
• Only minor effects were observed on redox potential.
• pH was lowest for treatments including *A. taxiformis*.

## Graphical Abstract

**Figure:**
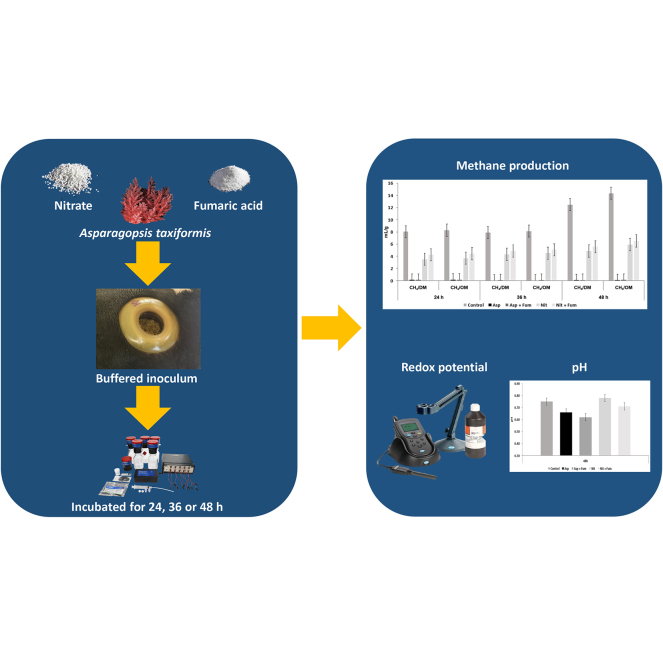
**Summary:** Implementation of potent methane mitigation feed additives is often associated with increased hydrogen production in the rumen of dairy cows. This may indirectly impair rumen fermentation and hence negatively affect animal production performance. We investigated the effect of a known hydrogen sink, fumaric acid, supplemented in combination with either *Asparagopsis taxiformis* or nitrate, as methane mitigation inhibitors, on in vitro gas production, degradability, pH, and redox potential in fermented rumen fluid. Both *A. taxiformis* and nitrate reduced methane production; however, detectable hydrogen levels were found only when *A. taxiformis* was supplemented. Hydrogen production was not affected by the inclusion of fumaric acid. Additionally, a linear negative relationship was found between in vitro pH and redox potential.

The use of feed additives to reduce enteric methane (CH_4_) production from cattle has become an intensively investigated research area during the past decade. The red macroalgae, *Asparagopsis* spp., and nitrate are effective CH_4_ mitigation feed additives both in vitro and in vivo (*Asparagopsis*: Kinley et al., 2016; Machado et al., 2018; Roque et al., 2019; nitrate: Olijhoek et al., 2016; Liu et al., 2017). However, their modes of action are believed to differ. It is suggested that halogenated compounds in *Asparagopsis* spp. inhibit the function of specific enzymes involved in the methanogenesis in the rumen (Glasson et al., 2022), while nitrate reduces CH_4_ emission through capture of the substrate hydrogen (H_2_) during its reduction in the rumen to ammonia via 2 energetically favorable reactions (Ungerfeld and Kohn, 2006). Another suggested mode of action of supplementing nitrate is the direct toxic effect of nitrite on methanogenic microorganisms, resulting in an inhibited microbial activity of these (Liu et al., 2017). The latter function may help to explain observations of elevated rather than reduced H_2_ emissions in in vivo studies, when CH_4_ emission was inhibited by dietary nitrate supplementation (van Zijderveld et al., 2011; Olijhoek et al., 2016). Increased H_2_ emissions have also been observed in a study where *Asparagopsis armata* was fed to dairy cows (Roque et al., 2019), which appears to be a logical consequence of the direct enzymatic inhibition of methanogenesis. However, a surplus of electron donors from H_2_ accumulation may indirectly suppress bacterial fermentation and microbial synthesis, and hence negatively affect animal performance (McAllister and Newbold, 2008). This may limit the implementation of otherwise potent feed additives to reduce CH_4_ emission. Indicators of rumen environment and microbial dynamics are, among others, ruminal pH and redox potential.

Reduction of fumaric to succinic acid and further decarboxylation to propionic acid is another H_2_ consuming pathway normally present in the rumen. Fumaric acid has previously been investigated as a CH_4_ mitigating additive, based on its capability to redirect H_2_ away from CH_4_ production (Bayaru et al., 2001; Newbold et al., 2005). However, to our knowledge, the role of fumaric acid as an electron acceptor under conditions with high H_2_ pressure facilitated by supplementation of either *A. taxiformis* or nitrate has not yet been investigated. Therefore, the present study was undertaken to investigate the effects of fumaric acid in combination with either *A. taxiformis* or nitrate on in vitro gas production, pH, and redox potential in an in vitro system simulating rumen fermentation. To investigate possible changes in fermentation patterns, the effects were evaluated after 24, 36, and 48 h of anaerobic fermentation.

*Asparagopsis taxiformis* was collected at Nelly Bay (19.2°S, 146.8°E), Magnetic Island, Australia (bromoform content: 4.2 mg/g DM). After harvest, the biomass was stored at −80°C until freeze-drying. Dietary nitrate [5Ca(NO_3_)_2_∙NH_4_NO_3_∙10H_2_O; 75.7% NO_3_; SilvAir] was provided by Cargill Inc., and fumaric acid was purchased from Bartek Ingredients Inc.

In 2 separate runs, 0.5 g of a standard feed, corn silage (ash: 3.54%; CP: 7.77%; NDF: 32.9%; starch: 35.1% on a DM basis), was added to Duran bottles (capacity: 132 ± 1.1 mL). To the corn silage there was further addition of 0.05 g of nitrate (**Nit**), 0.05 g of *A. taxiformis* (**Asp**), 0.05 g of nitrate + 0.05 g of fumaric acid (**Nit+Fum**), or 0.05 g of *A. taxiformis* + 0.05 g of fumaric acid (**Asp+Fum**; Table 1). Corn silage without any additives served as control. In 2 separate runs, 9 replicates per treatment were randomly distributed into 3 equal-sized blocks. After 24, 36, and 48 h, 3 replicates per treatment (one per block) were stopped in each run.

**Table 1:** Overview of treatments and content of OM and ash as a percentage of DM

Item	Corn silage	*Asparagopsis taxiformis*	Nitrate	Fumaric acid
Treatment^1^ (g)				
Control	0.5			
Asp	0.5	0.05	—	—
Asp+Fum	0.5	0.05	—	0.05
Nit	0.5	—	0.05	—
Nit+Fum	0.5	—	0.05	0.05
Chemical composition				
OM (%)	96.5	40.6	74.0	99.9
Ash (%)	3.5	59.4	26.0	0.01

1 Control = corn silage; Asp = *Asparagopsis taxiformis*; Asp+Fum = *Asparagopsis taxiformis* + fumaric acid; Nit = nitrate; Nit+Fum = nitrate + fumaric acid.

Rumen fluid from 3 rumen cannulated nonlactating Danish Holstein cows was sampled approximately half an hour before the morning feeding. The handling and care of the cows complied with the guidelines set out by the Danish Ministry of Environment and Food (Act No. 2028, 2020) concerning animal experimentation and care of animals under studies. The cows were fed 2 equal-sized meals of a diet consisting of grass/clover hay, barley straw, and a pelleted concentrate mixture (39.6% barley grain, 39.9% oat grain, 10% soybean meal, 3% rapeseed meal, 3% sugar beet molasses, and 4% of a commercial mineral mixture per kg of fresh mixture) at maintenance level, which amounted to 7.7 kg of DM daily (with 13.7% starch, 13.9% CP, 24.1% NDF, and forage-to-concentrate ratio of 69:31 on a DM basis).

At collection of rumen fluid, rumen liquid and particles were filtered through one layer of cheesecloth into preheated vacuum flasks.

Buffer solution included buffer, redox indicator, reducing agent, and macro and micro mineral solutions and were prepared as described by Menke and Steingass (1988). Rumen fluid was added to the buffer solution in a ratio of 1:2 and was continuously flushed with N_2_ to ensure anaerobic conditions of the inoculum (initial solution pH = 8.01). Ninety milliliters of inoculum was added to each Duran bottle, and the bottle headspace was flushed with N_2_ before a wireless, fully automated pressure sensor module from Ankom^RF^ Gas Production System (Ankom Technology) was fitted on top. The bottles were incubated in an incubator shaker (New Brunswick Excella E25R, Eppendorf) at 38.5°C and 50 oscillations per minute. Pressure changes in the headspace of the bottles during the incubations were continuously measured as a difference with respect to concurrently measured atmospheric pressure. When the pressure inside the bottle reached 5.17 kPa above ambient pressure, gas was released with a 250 ms vent opening into a gas-tight FlexFoil bag (1 L; SKC Ltd.), attached to the pressure sensor module.

After 24, 36, or 48 h of incubation, subsets of bottles representing each sample type were stopped, and pH and redox potential in the fermented liquid were measured immediately. The pH was measured using a digital pH-meter (Meterlab PHM 220, Radiometer), and redox potential was measured after 1 min of stabilizing using a redox meter with a platinum electrode (Intellical MTC101 ORP/redox electrode, Hach). The bottle content was filtered through F57 fiber bags (Ankom Technology) for determination of amount of undegraded feed residues.

From each gasbag, 10 mL of gas was extracted using a gas-tight syringe with Luer lock (Hamilton Bonaduz AG) and transferred into evacuated GC vials (Labco Limited) for analysis of CH_4_ and H_2_. Gas samples were analyzed within 1 wk after sampling.

*Asparagopsis taxiformis* and corn silage were freeze-dried and ground on a 1- and 2-mm screen, respectively (Ultra Centrifugal Mill ZM 200, Verder Scientific). Nutrient content of corn silage was analyzed using near-infrared spectroscopy at a commercial feed testing laboratory (Eurofins Steins A/S, Holstebro, Denmark). Contents of DM and OM in the samples were determined by oven-drying the samples at 103°C for 19 h and used for calculation of amount of DM and OM added to bottles. Degradable DM (**dDM**; %) was calculated as 100 × [(total DM feed + additives added to incubation bottles) − (DM residue after incubation)]/(total DM feed + additives added to incubation bottles). Degradable OM (**dOM**) was determined similarly to dDM. Ash content in samples and residues was determined by combustion at 525°C for 6 h. Ash content in nitrate was based on a technical calculation due to its explosive properties, where it was assumed that calcium oxide would be the only element left after combustion (Table 2).

**Table 2:** Methane (CH_4_) and hydrogen (H_2_) production per gram of incubated DM and OM after 24, 36, and 48 h of incubation

Parameter	Treatment^1^					SEM	*P*-value
	Control	Asp	Asp+Fum	Nit	Nit+Fum		
24 h							
CH_4_/DM (mL/g)	8.04^a^	0.131^c^	0.127^c^	3.52^b^	4.25^b^	0.798	<0.001
CH_4_/OM (mL/g)	8.23^a^	0.143^c^	0.139^c^	3.72^b^	4.46^b^	0.813	<0.001
H_2_/DM (mL/g)	—	2.02	2.11	—	—	0.312	0.83
H_2_/OM (mL/g)	—	2.20	2.30	—	—	0.342	0.85
36 h							
CH_4_/DM (mL/g)	9.05^a^	0.0560^c^	0.0778^c^	4.32^b^	4.87^b^	0.982	<0.001
CH_4_/OM (mL/g)	9.33^a^	0.0553^c^	0.0845^c^	4.56^b^	5.11^b^	1.02	<0.001
H_2_/DM (mL/g)	—	0.822	1.36	—	—	0.572	0.42
H_2_/OM (mL/g)	—	0.896	1.47	—	—	0.620	0.43
48 h							
CH_4_/DM (mL/g)	12.5^a^	0.0712^c^	0.108^c^	4.87^b^	5.58^b^	0.468	<0.001
CH_4_/OM (mL/g)	14.4^a^	0.0834^c^	0.1240^c^	5.96^b^	6.57^b^	0.824	<0.001
H_2_/DM (mL/g)	—	1.24	1.76		—	0.485	0.46
H_2_/OM (mL/g)	—	1.39	1.91		—	0.530	0.47

a–c Values within a row with different superscripts differ (*P* < 0.05).

1 Control = corn silage; Asp = *Asparagopsis taxiformis*; Asp+Fum = *Asparagopsis taxiformis* + fumaric acid; Nit = nitrate; Nit+Fum = nitrate + fumaric acid.

Concentrations of CH_4_ in gas were determined using GC (Trace 1310, Thermo Scientific) with a split/splitless injector at 150°C and a TCD at 250°C. A 30 m × 0.25 mm × 8 µm Rt-Q-BOND column (Restek) was used with helium as carrier gas at 1.7 mL/min flow. The GC oven was programmed with a ramped temperature starting at 50°C for 1.75 min and then increased from 50 to 150°C at 20°C/min. Concentration of H_2_ was determined using a GC (Trace 1310, Thermo Scientific) with a split/splitless injector at 150°C and a TCD at 200°C. A 30 m × 0.53 mm × 50 µm TG-BOND Msieve 5A column (Thermo Scientific) was used with nitrogen as carrier gas at 4 mL/min flow. The GC oven was programmed with a ramped temperature starting at 40°C for 5 min and then increased from 40 to 150°C at 20°C/min. Quantification was performed using standard curves with standard gases in concentrations ranging from 250 to 250,000 ppm for CH_4_ and from 1,000 to 500,000 ppm for H_2_.

Cumulative pressure of gas produced during fermentation was converted to milliliters of gas produced at standard temperature and pressure (IUPAC, 2014), assuming that the ideal gas law applied to the produced gases (predominantly CO_2_, CH_4_, and H_2_). Methane and H_2_ productions (mL) were calculated from the total gas production after the incubation and concentration of the gases (%). Total gas production, degradable DM, and dOM response parameters were blank corrected before the statistical analyses. Apparent efficiency of CH_4_ reduction was calculated as the measured reduction in CH_4_ relative to the potential reduction, based on stoichiometry.

The statistical analyses were conducted in R 4.1.2 (R Core Team, 2020) for each data set according to incubation time. Data are presented in tables as estimated marginal means and standard error of means. Effects of the response parameters were analyzed with a linear mixed model containing the fixed effect of treatment and random effect of experimental run. Shapiro-Wilk test and QQ-plots were used for evaluation of the normality of the residuals, whereas Bartlett's test and residual plots were used to evaluate the homogeneity of the variance. Two-way ANOVA was used to compute the *P*-values for the fixed effect of treatment. Differences between estimated marginal means were evaluated using Tukey's method for comparison. Statistical significance was declared when *P* ≤ 0.05, and statistical tendencies were declared when 0.05 < *P* ≤ 0.10.

In the Nit treatment, CH_4_ per gram of incubated DM was reduced by 56.2%, 52.3%, and 63.3% after 24, 36, and 48 h of incubation, respectively (Table 2). The effect was similar for CH_4_ per gram of incubated OM. These reductions were comparable to reductions achieved in an in vitro study where increased H_2_ emissions were also found when nitrate was supplemented (Guo et al., 2009). In our study, H_2_ production did not reach detectable levels in Nit or Nit+Fum samples. Therefore, it was not possible to evaluate the effect of combining nitrate and fumaric acid on H_2_ production. However, CH_4_ per gram of DM was not reduced any further when fumaric acid was included in combination with nitrate, compared with the Nit treatment. Stoichiometrically, 1 mol of nitrate would reduce CH_4_ by 1 mol. In our study, we supplemented nitrate at a dose of 6.9% (69 mg/g DM). This should yield a theoretical reduction in CH_4_ of 24.9 mL/g DM. After 48 h of incubation, CH_4_ per gram of DM was reduced from 12.5 mL/g DM to 4.87 mL/g DM, equivalent to 7.63 mL/g. Thus, we achieved an apparent efficiency of nitrate of 30.6%. We postulate that the lack of elevated H_2_ when nitrate was supplemented could be due to an incomplete reduction of nitrate to ammonia, since all available dissolved H_2_ was used for nitrate reduction or other competing pathways. However, CH_4_ production was not completely eliminated upon nitrate addition. Based on changes in Gibbs free energy (ΔG), nitrate reduction to ammonia is energetically more favorable than methanogenesis (Ungerfeld and Kohn, 2006). In contrast, van Lingen et al. (2016) suggested that classical thermodynamic functions may not fully apply for rumen fermentation reactions as the system is not near equilibrium, which indicates that other factors than changes in Gibbs free energy may determine the fate of H_2_.

*Asparagopsis taxiformis* with or without fumaric acid significantly reduced CH_4_ production compared with control at all incubation times by at least 98%. The reduction in CH_4_ when A. *taxiformis* was added in our study was similar to the reductions reported in other studies (Kinley et al., 2016; Machado et al., 2018). Certain ruminal bacteria can reduce fumaric acid to succinic acid, which is a precursor for propionic acid. This process uses H_2_ as electron donor, thus limiting availability of H_2_ for methanogenesis. However, when the partial pressure of H_2_ is low, the affinity of these ruminal bacteria for H_2_ is lower than that of methanogens (Asanuma et al., 1999). The Asp and Asp+Fum treatments were the only ones resulting in H_2_ levels above the detection level (0.05%; Table 2). There was no effect of adding fumaric acid to *A. taxiformis* on production of H_2_. When methanogenesis was eliminated, a large amount of H_2_ was available for succinate-forming bacteria if the population is sufficiently large. As these bacteria are expected to use H_2_ with fumaric acid as final electron acceptor (Asanuma et al., 1999), we expected a lower H_2_ production in Asp+Fum compared with Asp. We speculate that the dissolved H_2_ concentration never reached sufficiently high levels needed for those bacteria, as the gas is continuously released from the Ankom in vitro at a low level of pressure in the headspace. However, based on stoichiometric calculations, only a minor part (~2.5%) of the theoretical excess of H_2_ can be accounted for by measured increased production of H_2_, assuming that the production of 1 mol CH_4_ consumes 4 mol H_2_ (Boadi et al., 2004). This could imply that some H_2_ is redirected into other products such as propionate, butyrate, valerate, caproate, lactate, formate, or primary alcohols, which have not been investigated in this study.

In the rumen, the methanogens indirectly sustain the fermentation process by removing H_2_ and thereby reducing any negative feedback inhibition (McAllister and Newbold, 2008). The severe inhibition of methanogenesis by Asp and Asp+Fum did not affect the degradability of DM and OM at any incubation time compared with control (Table 3). These results agree with results reported in other in vitro studies investigating *A. taxiformis* (Kinley et al., 2016; Machado et al., 2018). In a study by Roque et al. (2019), DMI was reduced by 38.0%, when 1% of *A. armata* (on an OM basis) was included in the diet of dairy cows. A similar effect on feed intake was found by Roque et al. (2021) when *A. taxiformis* was supplemented to beef steers. In both studies, a large increase in H_2_ emission was also observed (Roque et al., 2019, 2021), which may have hampered fermentation and thus a negative effect on DMI. In the current study, using the Ankom in vitro system, any possible negative effect of increased H_2_ pressure on fermentation (Janssen, 2010) may not be measurable due to the gas being released from the bottles when the pressure reached a level of only 5.17 kPa above ambient pressure. Partial pressure of the major gases (CO_2_ and CH_4_) in the rumen headspace has been reported by van Lingen et al. (2017) to exceed this level considerably. In our study, a lower pressure of H_2_ in the headspace of the bottles might have made it impossible for H_2_ to re-dissolve into the liquid phase, as assumed by Wang et al. (2013). The discrepancy between the effects of *Asparagopsis* spp. on DMI in vivo versus feed degradability in vitro may be due to the differences in H_2_ pressure in these systems. The greater H_2_ pressure in vivo may result in a greater negative feedback on rumen fermentation.

**Table 3:** Degradability of dry matter (dDM) and organic matter (dOM), pH, and redox potential after 24, 36, and 48 h of incubation

Parameter	Treatment^1^					SEM	*P*-value
	Control	Asp	Asp+Fum	Nit	Nit+Fum		
24 h							
dDM (%)	48.5^ab^	50.2^a^	52.4^a^	41.1^b^	47.6^ab^	2.18	<0.01
dOM (%)	48.3^ab^	50.5^a^	50.4^a^	44.4^b^	49.8^a^	1.29	0.01
Redox (mV)	−350	−349	−346	−351	−340	5.23	0.16
pH	6.83^a^	6.71^c^	6.67^d^	6.85^a^	6.77^b^	0.0365	<0.001
36 h							
dDM (%)	67.2^a^	66.0^ab^	67.6^a^	61.2^b^	63.4^ab^	2.08	<0.01
dOM (%)	63.9^ab^	63.8^ab^	65.9^a^	60.3^b^	63.5^ab^	1.80	0.05
Redox (mV)	−346	−346	−345	−350	−350	4.33	0.65
pH	6.77^b^	6.67^d^	6.62^e^	6.79^a^	6.74^c^	0.0199	<0.001
48 h							
dDM (%)	72.9^a^	69.3^ab^	70.0^a^	64.0^b^	68.9^ab^	1.55	<0.001
dOM (%)	70.1	68.3	69	65.8	70.4	1.81	0.06
Redox (mV)	−343^ab^	−329^a^	−330^a^	−350^b^	−333^a^	6.43	<0.01
pH	6.75^a^	6.66^c^	6.62^d^	6.78^a^	6.71^b^	0.01605	<0.001

a–d Values within a row with different superscripts differ (*P* < 0.05).

1 Control = corn silage; Asp = *Asparagopsis taxiformis*; Asp+Fum = *Asparagopsis taxiformis* + fumaric acid; Nit = nitrate; Nit+Fum = nitrate + fumaric acid.

In the Nit treatment, dDM was lower after 36 and 48 h compared with control (−8.9 and −12.2%, respectively). This might be caused by a toxic effect on the fermentative bacterial population (Liu et al., 2017), although no negative effect of nitrate supplementation on nutrient digestibility was reported in vivo (Olijhoek et al., 2016). At all incubation times, pH was significantly lower for Asp and Asp+Fum compared with the control, and also lower for Asp+Fum compared with Asp. This might indicate a greater total VFA production, and thereby decreased pH, as *A. taxiformis* and fumaric acid could serve as substrate for fermentation. The greater pH and lower redox potential after 48 h for Nit compared with control could indicate a shift in fermentation patterns toward less propionate proportion, as a review by Huang et al. (2018) reported a positive correlation between redox potential and propionate proportion, associated with a negative correlation between the redox potential and pH. This could also explain the lower redox potential in Nit compared with Nit+Fum, since fumaric acid is a precursor for propionate synthesis in the rumen in a H_2_ consuming process.

An unpublished in vivo study (M. Thorsteinsson, P. Lund, M. R. Weisbjerg, S. J. Noel [Aarhus University, Tjele, Denmark], A. A. Schönherz [Aarhus University, Tjele, Denmark], A. L. Frydendahl Hellwing [Aarhus University, Tjele, Denmark], H. H. Hansen [University of Copenhagen, Frederiksberg, Denmark], and M. O. Nielsen) reported a linear negative relationship (R^2^ = 0.86) between ruminal pH and redox potential when the methanogenesis was inhibited in dairy cows by a novel feed additive, currently under patenting. Friedman et al. (2017) observed a similar relationship in vitro. Such a relationship is expected as many reactions caused by the ruminal microbiota involve protons in addition to the transfer of electrons, and according to the Nernst equation redox potential and pH are mutually dependent (Dijkstra et al., 2020). This agrees with the data from our study, as a linear negative correlation between pH and redox potential (R^2^ = 0.73) occurred across all incubation times and treatments.

Data from this experiment indicate that the settings used in the Ankom in vitro system in this experiment do not completely mimic the complicated dynamics of gaseous exchange in the ruminal in vivo environment. In in vitro systems, it is possible to observe similar directions of change in the gas production as in in vivo studies, but not all aspects of rumen thermodynamics and kinetics. Applying a greater release pressure in the in vitro system could be an approach to more closely imitate the rumen environment under severe inhibition of the methanogenesis and accumulation of H_2_. Additionally, a dose-response approach might be valuable in such investigations in the future.

In conclusion, adding *A. taxiformis* and nitrate alone to corn silage in an in vitro system reduced CH_4_ production. However, only supplementation of *A. taxiformis* resulted in detectable levels of H_2_ production when CH_4_ was inhibited. Additionally, combining fumaric acid with *A. taxiformis* did not decrease the elevated H_2_ production. *Asparagopsis taxiformis* and the combination of *A. taxiformis* and fumaric acid reduced CH_4_ production by at least 98% at all incubation times, whereas nitrate alone or in combination with fumaric acid reduced CH_4_ by 52% to 63%, respectively, compared with the control. A negative correlation between pH and redox potential was found. For future in vitro studies investigating the potential of fumaric acid as hydrogen sink during CH_4_ inhibition, dose-response approaches along with technical adjustments of the Ankom system are relevant considerations.
